# Prevention of the Development or Use of Biological Weapons

**DOI:** 10.1089/hs.2016.0096

**Published:** 2017-02-01

**Authors:** Gigi Kwik Gronvall

Preventing the development and use of biological weapons should continue to be a top priority for the nation. There are fundamental issues that make prevention difficult, however. The knowledge, materials, and technologies needed to make and use a biological weapon are readily accessible around the world. Pathogens are ubiquitous in nature and can be found in hospital and research laboratories, scientific culture collections, and in sick people and animals everywhere. It is now possible to synthesize pathogens from scratch, particularly viruses, with technologies that are inexpensive and globally available. The skills and equipment for making a biological weapon are largely the same as those required for progress in medicine, agriculture, and other fields and are required for future economic prosperity for the nation, so they cannot be locked away. Efforts that might be useful in deterring terrorist groups will be different than those targeted toward nation states—and *every* nation state is presumed to have the technical and financial resources to have a biological weapons program should they choose to embark on one.

In spite of these challenges, the US government has options for increasing the likelihood that biological attacks can be prevented, through maintaining international norms and improving surveillance systems, deterring potential adversaries by demonstrating a strong national response, developing better forensic analysis, generating better intelligence, and implementing sensible security practices for legitimate scientists.

## Recommendations

➢ **The US government should continue to strongly support the Biological Weapons Convention and other international efforts that both prevent terrorism and promote the development of a global public health infrastructure.**

The cornerstone of biological nonproliferation strategies is the Convention on the Prohibition of the Development, Production and Stockpiling of Bacteriological (Biological) and Toxin Weapons and on Their Destruction, commonly known as the Biological Weapons Convention (BWC). The BWC is the first agreement among nations that declared an entire category of weapons to be off limits. The moral force of the treaty has not prevented all of its signatories from developing biological weapons: For example, the Soviet Union, a signatory to the convention, established an enormous secret bioweapons program during the Cold War, and there are some current signatories that are highly likely to have offensive biological weapons programs. However, no country openly goes against the international norm to display an offensive biological weapons capability.

This prohibition against biological weapons development should continue to be strengthened with vigorous US support to promote universal adoption of the treaty and with implementation support to other signatories. Other international agreements intended to prevent terrorism, such as UN Resolution 1540, and measures such as the Global Health Security Agenda, which focus donor attention on areas where the global public health infrastructure needs to be strengthened, also should be actively promoted by the US government. The devastating Ebola outbreak in Guinea, Sierra Leone, and Liberia that killed 11,310 people in 2014-15 should be seen as a harbinger of what could happen again should public health infrastructure not be maintained throughout the world, and what could easily result from a deliberate bioattack.

➢ **The United States should strengthen deterrence of biological weapons by expanding our ability to reduce the consequences of such attacks and by increasing investments in surveillance and microbial forensics capacities.**

The consequences of a biological attack can be reduced significantly by a rapid medical response to detect, treat, and provide appropriate medical care. If the United States has the demonstrated capacity to seriously limit the consequences of a biological weapons attack through a rapid and effective response, this may deter some adversaries from pursuing a biological attack. In addition to this form of deterrence, the United States should press ahead with efforts to strengthen its ability to perform rapid surveillance, as well as sophisticated analysis of microbial forensics. The nation needs to develop the strongest possible scientific capacity to trace back a pathogen to its natural or laboratory origin—an important part of attributing an attack to its source.

➢ **The United States should increase its support for private sector efforts to screen gene synthesis orders.**

Gene synthesis companies provide services that support medical research, as well as biotechnology and pharmaceutical product development. However, these services could shorten timelines for developing a biological weapon if the genetic material that encodes a pathogen is made to order. In response to this threat, a number of international gene synthesis companies have already formed a compact to screen orders. Their screening exceeds the guidelines put forward by the Department of Health and Human Services in 2010. However, these screening controls are becoming less valuable: Not all companies screen orders, there is a time/money cost advantage that nonadherents to the guidelines benefit from, and screening is becoming more expensive to companies. As the costs to produce genes are dropping, the costs of screening have remained constant, so the screening is becoming more of a burden and inhibitory to the companies' competitiveness.

The US government can do more to support this screening mechanism to preserve this preventative measure in the years ahead. The United States should work to increase the number of companies that perform gene screening, promote awareness of companies' codes of conduct, and encourage providing those companies with a means to report a suspicious order.

Finally, there is a need to research the effectiveness of this preventative measure, and how to improve it, as there are no publicly available data about how valuable the sequence screening is in stopping misuse. It could be that it is customer screening, and not sequence screening, that is the most valuable part of the security screening process, and research should be funded to examine its effectiveness and how best to improve screening.

➢ **Intelligence collection should be strengthened to identify and interdict would-be bioterrorists before an attack.**

In 2005, the Commission on the Intelligence Capabilities of the United States Regarding Weapons of Mass Destruction stated that the biological weapons threat is the “mass casualty threat the [intelligence community] is least prepared to face.” These conclusions were echoed by the 2015 Blue Ribbon Study Panel on Biodefense. Making progress will require placing a higher priority on US intelligence collection against biological threats, finding better ways to reward biological expertise and analytic skills in the intelligence community, building better coordination between the intelligence community and the scientific and health communities, and providing more resources to the intelligence community for these efforts.

➢ **US efforts to control legitimate science that may lower barriers to biological weapons development are limited. Nonetheless, an educational and awareness-raising campaign should be part of scientific training.**

Over the past 15 years, the US government has taken numerous actions to prevent legitimate science from lowering barriers to biological weapons development by nefarious actors. The Department of Health and Human Services (HHS) advisory committee, the National Science Advisory Board for Biosecurity (NSABB), defined such dual-use research of concern (DURC) as “life sciences research that, based on current understanding, can be reasonably anticipated to provide knowledge, information products, or technologies that could be directly misapplied to pose a significant threat with broad potential consequences to public health and safety, agricultural crops and other plants, animals, the environment, materiel, or national security.” US scientists who work with regulated pathogens are currently required to screen their research for issues related to dual-use research of concern. However, it is hard to know, without extraordinary insight into the minds and plans of nefarious actors, how a particular piece of scientific knowledge may be misused. It is also a challenging process to balance the risks of publication and subsequent misuse along with the potential for a more positive outcome.

It will be difficult to manage the “dual-use” problem without also inappropriately limiting science, with uncertain effects on increasing security. Yet, all scientists should know that their work could be misused, and there should be a process to evaluate such research. For particularly problematic studies, scientists should be able to explain the steps that have been taken to minimize that risk, and to explain the importance of what they are doing.

